# Supplementary material for: Accurate computation of ionic concentrations in the synaptic cleft requires the full Poisson–Nernst–Planck (PNP) equations
Source: PLoS Comput Biol. 2026 May 28;22(5):e1014341. doi: 10.1371/journal.pcbi.1014341 (PMC13235942; doi:10.1371/journal.pcbi.1014341)
Supplement: Attachment — Submitted filename: response.pdf [file pcbi.1014341.s003.pdf]

Response to reviewer comments

Reviewer #1

This is a nice paper showing the significant consequences of neglecting electrical field effects on ion diffusion in a synaptic cleft of a glutamatergic AMPA synapse. The authors compare purely diffusive model with the full Poisson-Nerst-Planck model of coupled diffusion of individual ionic species with the Poisson equation on the electric potential.

The paper is well written, the models and simulations are clearly defined and described. Results are adequately illustrated, even if the individual figure panels are rather small, I think they are sufficient to represent the individual effects that are observed and discussed.

Authors' response:

Thank you for reading the paper and for your encouraging remarks.

I have not seen errors. Some minor comments:

- I would finish the first sentence of the second paragraph in the Introduction with "for glutamatergic AMPA synapse" etc., I had a moment of hesitation on the generality of the announced research. I understand the potential for translation, of course.

- in Table 1 and 2 do we need so many significant digits? In particular, how valid is providing the concentration of intracellular Cloride with 7 significant digits?

- In 3.4.3 "fluxes into or out of the cleft has" -> have?

- In 3.4.4 "fluxes into or out of the cleft causes" -> cause?

Authors' response:

We have clarified the scope in the first sentence of the second paragraph of the Introduction as suggested, and corrected the grammatical errors in Sections 3.4.3 and 3.4.4. Regarding Tables 1 and 2, the physical constants in Table 1 are taken from standard reference values, and the concentrations in Table 2 were chosen to ensure initial electroneutrality. We therefore retained the required precision for values where rounding would introduce a small but artificial net charge. To make this clear, we have added the following note to Table 2:

Some concentration values are reported with extra digits to ensure initial electroneutrality of the intracellular and extracellular solutions.

Since the main point of this paper is that the effects of the electric field are often comparable in size to the effects of diffusion, so the PNP is more realistic, I have a question on the realism of the model, specifically, how realistic is the continuous description of this process.

Given that a single synaptic vesicle will have several thousands glutamate molecules, and given the spatial precision of the figures, consider coarse-graining on the order of 10um or 1um per side. For cleft of 20um that would give us a partition into 7200 boxes. For a finer partition of 1um we would have 7.2 mln. boxes. That would give us very sparse occupation with Glutamate particles. So I wonder if molecular dynamics in the end is not more adequate in this context? I realize a comparison of PNP with MD simulations is a different paper, but I think the paper would benefit from a brief discussion of the effects of low particle numbers in the cleft on the realism of those results and possible relation to MD simulations.

Authors' response:

We agree that the continuum interpretation of PNP becomes less direct when the number of particles is small. We have therefore added a short discussion of this issue to the Limitations section of the manuscript. The added text clarifies that PNP should be interpreted as a continuous, mean-field approximation of an underlying discrete stochastic process, and that particle-based stochastic or molecular dynamics methods may be more appropriate when individual-particle fluctuations are central to the question:

Another limitation of the present approach is that PNP is a continuum, mean-field model, whereas vesicle release involves a limited number of glutamate molecules. At sufficiently fine spatial resolution, some local volumes would contain zero or one glutamate molecule, and the interpretation of a smooth concentration field therefore becomes less direct. The PNP solution should therefore be interpreted as an averaged description, rather than as an instantaneous particle realization. Particle-based stochastic or molecular dynamics models may be more appropriate when fluctuations in individual molecule positions are central to the question. However, to be comparable with the present model, such models would need to include five ion species present in the cleft, which would create a substantial computational challenge.

Unfortunately, the code for the paper is not provided so I was not able to inspect it. Making the code available would increase the impact of this work in the community. I encourage the authors to make their code public in a way which would encourage reproduction of those results.

According to the submission the code is available on Zenodo (DOI: 10.5281/zenodo.18952935). I was unable to find it.

Authors' response:

Since Zenodo records are versioned and less convenient to update during revision, we usually publish the archived software when the paper is accepted. However, we agree that making the code available during review is useful for inspection and reproducibility. We have therefore made the numerical code available on GitHub (see https://github.com/karolihj/PNP-synapse-FDM-2026).

Reviewer #2

The authors explore simulating the release of neurotransmitter from a single vesicle into an idealized synaptic cleft and the response of AMPA receptors in the postsynaptic membrane. They compare Poisson-Nernst-Plank (PNP) and pure diffusion models and conclude that inclusion of the electrical potentials in the PNP model generates significantly different predictions than the diffusion model which ignores electrical effects. This could have important ramifications for modeling synaptic neurotransmission, and thus is an important observation. As such, I am mostly enthusiastic about this work. There are a few points that need to be addressed prior to publication (see comments below for details). Primarily, they involve using a more realistic Markov model for AMPA receptors (this is a must), explaining what appears to be odd diffusional bias along certain dimensions, and reporting the comparison of PNP and diffusion models on the simulated postsynaptic current.

Authors' response:

Thank you.

1. The Markov model for AMPA receptors has a serious flaw in that it does not include desensitization. AMPA receptor currents are shaped by rapid activation and also rapid desensitization, which must be included for any realistic model of AMPA receptor behavior. This could also influence the final conclusion as to how much the PNP vs Diffusion models actually differ in their predictions for postsynaptic currents.

Authors' response:

We used the Markov model of Savtchenko and Rusakov in the original manuscript for simplicity, computational feasibility, and to keep the model close to their earlier investigation. We agree with the reviewer that this model does not include desensitization and is therefore limited as a model of AMPA receptor kinetics. We have therefore implemented the more comprehensive Markov model of Jonas, Major, and Sakmann, which includes desensitized states. The computations in the manuscript have been updated accordingly, and the comparison between the PNP and diffusion models is now based on this revised AMPA receptor model.

2. In Fig. 2, why does Glu appear to diffuse/disperse primarily in the y-direction and not z-direction? This seems odd to me. The depicted Glu transient appears almost like a laser that zaps across the synaptic cleft without much lateral diffusion. Can this be reconciled with spillover of neurotransmitter outside of the synaptic cleft which we know occurs? Some discussion as to what is driving this behavior should be included. The current explanation of this behavior simply describes the events without providing much explanation as to why they occur. Also, although there is more diffusion in the z-direction for the diffusion model, both Glu and ions still appear to primarily diffuse in the y-direction (see Fig. 3). Does this make sense? For the diffusion model, why should there be any bias for diffusion in y- vs z-directions, or the y- vs x- directions for that matter? As shown, even the diffusion model

appears to predict a broader laser zapping across the cleft. It makes me worry that there is something wrong with the simulation, although perhaps I am missing something. Either way, an explanation would be very helpful for most readers (and myself).

Authors' response:

The apparent directional bias in Figure 2 is a visualization effect caused by the aspect ratio used to display the y,z-slice. The physical extent of the domain in the z-direction is much larger than the cleft height in the y-direction. To display the full sheet, the axes are therefore not shown with equal physical scaling. This makes the glutamate transient appear more strongly directed in the y-direction than it is in physical coordinates. We have clarified the scaling in the caption and revised the text to explain that the apparent "laser-like" shape is a visualization effect, not a directional bias in the diffusion operator.

The following text has been included in the caption of Fig. 2 and Fig. 3:

Note that the y- and z-axes are not shown with equal physical scaling; the y-direction, corresponding to the cleft height, is enlarged relative to the z-direction to make the narrow cleft visible. The displayed domain is actually about 40 times longer in the z-direction than in the y-direction.

In addition, we have included a supplementary figure (Fig. S1) which shows the solutions with equal physical scaling of the axes.

3. Ultimately, what matters most for those interested in simulating synaptic communication is the postsynaptic response. Although the authors thorough description of the various in-cleft distributions of Glu and ions is certainly still highly relevant, I would ask them to report the simulated postsynaptic current through AMPA receptors, i.e., the EPSC, and to compare the predicted EPSC between PNP and diffusion models. Of course, this must be done with a physiologically realistic AMPA receptor Markov model that includes desensitization. This information is crucial for many to decide whether the added cost of the PNP model is worth it for their purposes.

Authors' response:

We would like to emphasize that the main aim of the paper is to clarify the difference between pure diffusion and full PNP electrodiffusion in the synaptic cleft. However, we agree that the postsynaptic current is an important derived quantity. We have therefore added computations of the AMPA-mediated EPSC for both the D and PNP models, using the revised AMPA receptor Markov model that includes desensitization. These results are now reported in the new Figure 5, and discussed in the new Section 2.4. We have also added a comment in the abstract and in the Discussion.

4. There are charges on the lipid head groups in the membrane bilayer. It is unclear to what extent this is accounted for or if it matters.

Authors' response:

We agree that fixed charges associated with lipid head groups may influence the local electric potential and ionic concentrations near the membrane. These charges are not included in the present model. Our membrane representation is therefore electrically neutral, except for the mobile ionic concentrations and the imposed transmembrane fluxes through AMPA receptors. Including fixed membrane surface charge would require additional assumptions about membrane composition and surface charge density, and is outside the scope of the present study. We have added this point to the limitations section:

Furthermore, the model does not include fixed surface charges associated with lipid head groups in the membrane bilayer. Such charges may influence the local electric potential and ionic concentrations close to the membrane. Representing this effect would require additional assumptions about membrane composition and surface charge density, and it is therefore left outside the present model.

5. It is unclear how easily the PNP solution can be extended to more realistic synaptic cleft geometries. A brief comment on this would be helpful.

Authors' response:

The PNP system can in principle be implemented in more realistic synaptic cleft geometries. The present finite-difference implementation was chosen because the idealized cuboid geometry gives direct control of the boundary conditions and the involved fluxes, and allows us to isolate the effect of replacing the D model by the full PNP system. More realistic geometries would require a numerical method suitable for complex domains, such as the finite element method. We have added a brief comment on this point to the Discussion. We are currently developing such a FEM-based implementation, but it is not ready and it is outside the scope of the present study. The following text has been added to the limitations section of the paper:

The geometry used here is idealized — a flat cleft with parallel pre- and postsynaptic membranes — primarily to isolate the effect of the governing equations from confounding geometric factors. The actual geometry of the synaptic cleft is more complex, and morphological details such as membrane curvature and cleft width variations are known to influence neurotransmitter dynamics, see, e.g., [24]. The PNP equations themselves are not restricted to the idealized cuboid geometry applied here, but more realistic synaptic cleft geometries would require a numerical method suitable for complex domains, such as the finite element method. Such extensions are possible, but are outside the scope of the present study.
